# Integrated analysis of endophytic fungal communities and metabolites reveals root rot-induced disruptions in *Monochasma savatieri*

**DOI:** 10.3389/fpls.2026.1752821

**Published:** 2026-05-13

**Authors:** Yuyin Zhang, Hua Dou, Chenlu Fan, Xuyu Chen, Jianhe Wei

**Affiliations:** 1 Hainan Provincial Key Laboratory of Resources Conservation and Development of Southern Medicine, Hainan Branch of Institute of Medicinal Plant Development, Chinese Academy of Medicinal Sciences & Peking Union Medical College, Haikou, Hainan, China; 2 Key Laboratory of Bioactive Substances and Resources Utilization of Chinese Herbal Medicine, Ministry of Education & National Engineering Laboratory for Breeding of Endangered Medicinal Materials, Institute of Medicinal Plant Development, Chinese Academy of Medical Sciences & Peking Union Medical College, Beijing, China

**Keywords:** endophytic fungi, *M. savatieri*, metabolomics, plant disease, root rot

## Abstract

**Introduction:**

*Monochasma savatieri* is a traditional Chinese medicinal herb valued for its anti-inflammatory and antioxidant properties. Due to the depletion of wild resources, artificial cultivation of this species has expanded rapidly in recent years. However, the emergence of root rot has become a significant threat, potentially altering the plant’s endophytic microbiota and chemical composition. Understanding these microbial and metabolic changes is critical for improving disease management and maintaining medicinal quality.

**Methods:**

High-throughput sequencing (HTS) was employed to characterize the endophytic fungal communities in both healthy and root rot-infected *M. savatieri* plants. In parallel, non-targeted metabolomics based on liquid chromatography-mass spectrometry (LC-MS) was conducted to profile and compare the aqueous extracts of the two groups, aiming to assess metabolic alterations induced by root rot.

**Results:**

HTS revealed that root rot significantly reduced the diversity and evenness of endophytic fungal communities, accompanied by distinct shifts in specific taxa, including *Saitozyma* and *Paraphoma*. Diseased plants exhibited higher fungal abundance but lower community stability. Metabolomic analysis demonstrated a clear separation between healthy and diseased samples, with 554 metabolites upregulated and 669 downregulated in diseased plants. These differentially accumulated metabolites were primarily involved in amino acid, lipid, and secondary metabolism, Pathway enrichment analysis further highlighting disruptions in tryptophan metabolism, ether lipid, and glycerophospholipid metabolism.

**Conclusion:**

Root rot substantially alters both the endophytic microbial structure and the metabolic landscape of *M. savatieri*, potentially compromising disease resistance and affecting the medicinal quality of the herb. The enrichment of specific amino acids and flavonoid metabolites in healthy plants suggests their possible involvement in plant defense mechanisms. Collectively, these findings provide a valuable foundation for future research on biological control strategies, metabolic regulation, and the establishment of quality standards for this medicinal species.

## 1 Introduction

*Monochasma savatieri* Franch. (Scrophulariaceae), commonly known as “Lu Rong Cao” in Chinese traditional medicine, is a medicinal herb whose dried aerial parts have long been used for clearing heat, relieving pain, cooling the blood, and stopping bleeding. Increasing pharmacological research has revealed its potential in anti-inflammatory, antioxidant, antitumor, and cardiovascular protective effects, making a key ingredient in the traditional Chinese patent medicine “Yanning Syrup” (Zhang et al., 2025). *M. savatieri* is primarily distributed in sandy mountainous regions and undergrowth in Jiangxi, Zhejiang, and Fujian provinces of China. However, due to overharvesting and habitat destruction, wild populations are facing depletion (Chen et al., 2021). To meet growing demand and ensure sustainable utilization, large-scale artificial cultivation has been initiated in areas such as Yichun, Jiangxi Province. Although cultivation reduces pressure on wild resources, it has also led to severe disease outbreaks, particularly root rot caused by soil-borne pathogenic fungi (Wang et al., 2024). Despite the increasing severity of this disease under cultivation, its impacts on the endophytic fungal community and metabolic profiles of M. savatieri remain poorly understood.

Endophytic fungi play a roles in maintaining plant health and enhancing disease resistance (Li et al., 2025). Understanding the composition and dynamics of fungal communities in diseased roots is essential for elucidating plant-pathogen interactions and improving disease management (Chen et al., 2024). High-throughput sequencing (HTS) has emerged as a powerful tool for studying microbial communities, enabling comprehensive profiling of microbial diversity without the limitations of traditional culture-based methods (Nizamani et al., 2023; González-Dominici et al., 2024). Applying HTS to analyze changes in the endophytic fungal community of M. savatieri under root rot stress can provide insights into the disease’s microecological impact and inform strategies for maintaining plant health (Anzano et al., 2022). In addition to its effect on microbial communities, root rot also alters host plant physiological and secondary metabolite composition, potentially affecting medicinal quality (Yu et al., 2025). Investigating these metabolic changes is therefore critical for understanding the disease’s influence on phytochemical profiles and providing scientific evidence for quality control (Cajka T. et al., 2014). Using Liquid Chromatography-Mass Spectrometry(LC-MS) combined with multivariate data analysis, this technique facilitates the identification of metabolic alterations at a global scale. LC-MS is particularly suited for analyzing a wide range of compounds with high sensitivity and separation efficiency, while GC-MS excels in detecting volatile metabolites with superior resolution (Anzano et al., 2022). Compared with conventional chemical analyses, non-targeted metabolomics can identify key differential metabolites and provide supporting data for unknown compound discovery, greatly expanding the depth and breadth of metabolic research.

Currently, studies on the effects of root rot disease on the metabolites of *M. savatieri* are limited. Given the close relationships among plant health, endophytic microbial communities, and host metabolic profiles, we hypothesized that root rot significantly disrupts the assembly of the endophytic fungal community in *M. savatieri*, which in turn leads to profound alterations in the secondary metabolism of the aerial parts. To test this hypothesis, we integrated high-throughput sequencing of endophytic fungi with non-targeted metabolomics of aerial tissues. This study aims to elucidate the microecological shifts and metabolic mechanisms associated with root rot, thereby providing a theoretical foundation for the development of biological control strategies and quality assurance in the cultivation of *M. savatieri*.

## 2 Materials and methods

### 2.1 Sample collection

Both healthy and root rot–infected *M. savatieri* plants were collected in July 2024 from the cultivation base of Bingchen Agricultural Technology Co., Ltd. (Yuanzhou District, Yichun City, Jiangxi Province, China). Disease status was assessed in the field based on typical root rot symptoms, including root browning, tissue decay, and stunted above-ground growth. Three healthy plants (designated as HS-1, HS-2, and HS-3) and three plants exhibiting typical root rot symptoms (designated as SS-1, SS-2, and SS-3) were selected as independent biological replicates. All samples were placed into sealed bags immediately after collection, transported to the laboratory, and stored at 4 °C until further processing.

### 2.2 Analysis of the fungi in *M. savatieri* using high-throughput sequencing

#### 2.2.1 Genomic DNA extraction and PCR amplification

Genomic DNA was extracted from tissues of healthy (HS-1, HS-2, HS-3) and root rot–affected (SS-1, SS-2, SS-3) *M. savatieri* plants using the cetyltrimethylammonium bromide (CTAB) method. DNA purity and concentration were assessed by agarose gel electrophoresis, and working solutions were diluted to 1 ng/μL. The fungal ITS1–ITS2 region was amplified using primers ITS1F (5′-CTTGGTCATTTAGAGGAAGTAA-3′) and ITS2R (5′-GCTGCGTTCTTCATCGATGC-3′), each containning with sample-specific barcodes. PCR amplification was performed in a 25 μL reaction mixture containing 12.5μL of Phusion^®^ High-Fidelity PCR Master Mix with GC Buffer (New England Biolabs), 0.2μM of each primer, and 10 ng of template DNA. The thermal cycling conditions were as follows: initial denaturation at 98 °C for 30 s; 35 cycles of 98 °C for 10 s, 55 °C for 30 s, and 72°C for 30 s; and a final extension at 72 °C for 5 min, following established protocols (Op De Beeck et al., 2014; Wang et al., 2018).

#### 2.2.2 Library construction and sequencing

Following DNA extraction, PCR amplification was carried out using universal primers fused with sequencing adaptors. The amplified products were purified, quantified, and normalized to construct sequencing libraries. Library quality was assessed prior to sequencing. Qualified libraries were sequenced using the Illumina NovaSeq 6000 platform. The raw image data generated by sequencing were converted into raw sequencing reads in FASTQ format, containing both sequence information and base quality scores.

#### 2.2.3 Data processing

Quality filtering: Raw reads were first quality-filtered using Trimmomatic v0.33. Primer sequences were identified and removed using Cutadapt 1.9.1, resulting in clean reads without primer contamination. Denoising (DADA2): The DADA2 plugin within QIIME2 2020.6 (Bolyen et al., 2019; Callahan et al., 2016) was used to denoise reads, merge paired-end sequences, and remove chimeras to obtain high-quality, non-chimeric reads amplicon sequence variants (ASVs). Subsequent analyses, including alpha diversity, beta diversity, differential abundance, and functional prediction, were conducted based on the ASV table.

Diversity analysis: Alpha diversity indices, including observed ASVs, Chao1, Shannon, Simpson, ACE, Goods_coverage, and PD_whole_tree, were calculated using QIIME. Rarefaction curves, rank-abundance curves, and species accumulation curves were generated in R (v2.15.3). Alpha diversity comparisons were also conducted using R software.

### 2.3 Non-targeted metabolomics analysis of aqueous extracts

#### 2.3.1 Sample preparation

Healthy samples were labeled as Lrc-H1, Lrc-H2, and Lrc-H3; while diseased samples were labeled as Lrc-S1, Lrc-S2, and Lrc-S3. For each sample, 2.0 g of dried *M. savatieri* from each group was weighed and extracted with 50.00 mL distilled water under reflux in a water for 2 hours. After cooling, the extracts were filtered.

The filtrates were transferred into pre-weighed evaporating dishes and concentrated to dryness in a water bath. The residue was dried at 105 °C for 3 hours, cooled in a desiccator for 30 minutes, and precisely weighed. The dried extracts were redissolved in solvent to a final concentration of 1 mg/mL. For LC-MS/MS analysis, 100 μL of each solution was transferred to a 1.5 mL centrifuge tube, vortexed for 15 minutes, and centrifuged at 12,000 rpm for 3 minutes at 4 °C. The supernatant was filtered through a 0.22 μm microporous membrane and transferred to autosampler vials.

#### 2.3.2 Chromatographic and mass spectrometry conditions

Chromatographic separation was performed on an ACQUITY UPLC HSS T3 column (1.8 μm, 2.1 mm × 100 mm, Waters) maintained at 40 °C. The mobile phase consisted of 0.1% formic acid in water (A) and 0.1% formic acid in acetonitrile (B), with a flow rate of 0.4 mL/min. The injection volume was 4 μL. The gradient program was as follows: 0–5 min, 95%–35% A; 5–6 min, 35%–1% (A); 6–7.5 min, 1% A; 7.5–7.6 min, 1%–95% (A); 7.6–10 min, 95% (A) (Jung et al., 2019).

#### 2.3.3 Data analysis

All samples were analyzed in triplicate. Principal component analysis (PCA), orthogonal partial least squares discriminant analysis (OPLS-DA), hierarchical cluster analysis (HCA), volcano plots, and KEGG pathway enrichment analysis were performed using the MetWare Cloud platform (https://www.metware.cn/), with statistical processing conducted in R (v3.5.1). Group differences were assessed using SAS^®^ 9.4 software, and differences were considered statistically significant when *P* < 0.05 (Chen et al., 2023). To account for potential false positives in high-dimensional non-targeted metabolomics data, multiple testing correction was applied where applicable, and differential metabolites were interpreted based on consistent trends across biological replicates and the stability of multivariate models. Given the exploratory nature of this study, the analyses aimed to identify major metabolic shifts associated with root rot rather than to establish definitive causal relationships.

## 3 Results

### 3.1 Comparison of endophytic fungal communities between healthy and root rot-infected *M. savatieri*

#### 3.1.1 Taxonomic classification of sequencing reads

Following quality control and denoising using the DADA2 pipeline, an ASV feature table was constructed. Sequencing reads were classified at seven taxonomic levels (Kingdom, Phylum, Class, Order, Family, Genus, and Species). The number of reads assigned to each taxonomic level for individual samples is summarized in **Table 1**. The values represent the total number of reads classified under each respective taxonomic level for the corresponding sample.

**Table 1:** Reads statistics of each sample.

Sample	Kingdom	Phylum	Class	Order	Family	Genus	Species
HS-1	45273	43660	27843	23865	22883	22398	14630
HS-2	70855	68022	66710	62947	62668	59142	48523
HS-3	63219	59634	57681	46211	43962	41652	29745
SS-1	63632	63046	58805	56868	56170	55022	35770
SS-2	66449	65096	62556	62409	61759	61271	40984
SS-3	61652	52519	49758	45711	45105	31943	21040

#### 3.1.2 Comparison of endophytic fungal alpha diversity between healthy and root rot-infected *M. savatieri* plants

To assess the impact of root rot disease on the internal fungal diversity of M. savatieri, four alpha diversity indices—Simpson, Chao1, PD whole tree, and Shannon—were compared between healthy (HS) and diseased (SS) plants using Student’s t-test.

**Figure 1:**
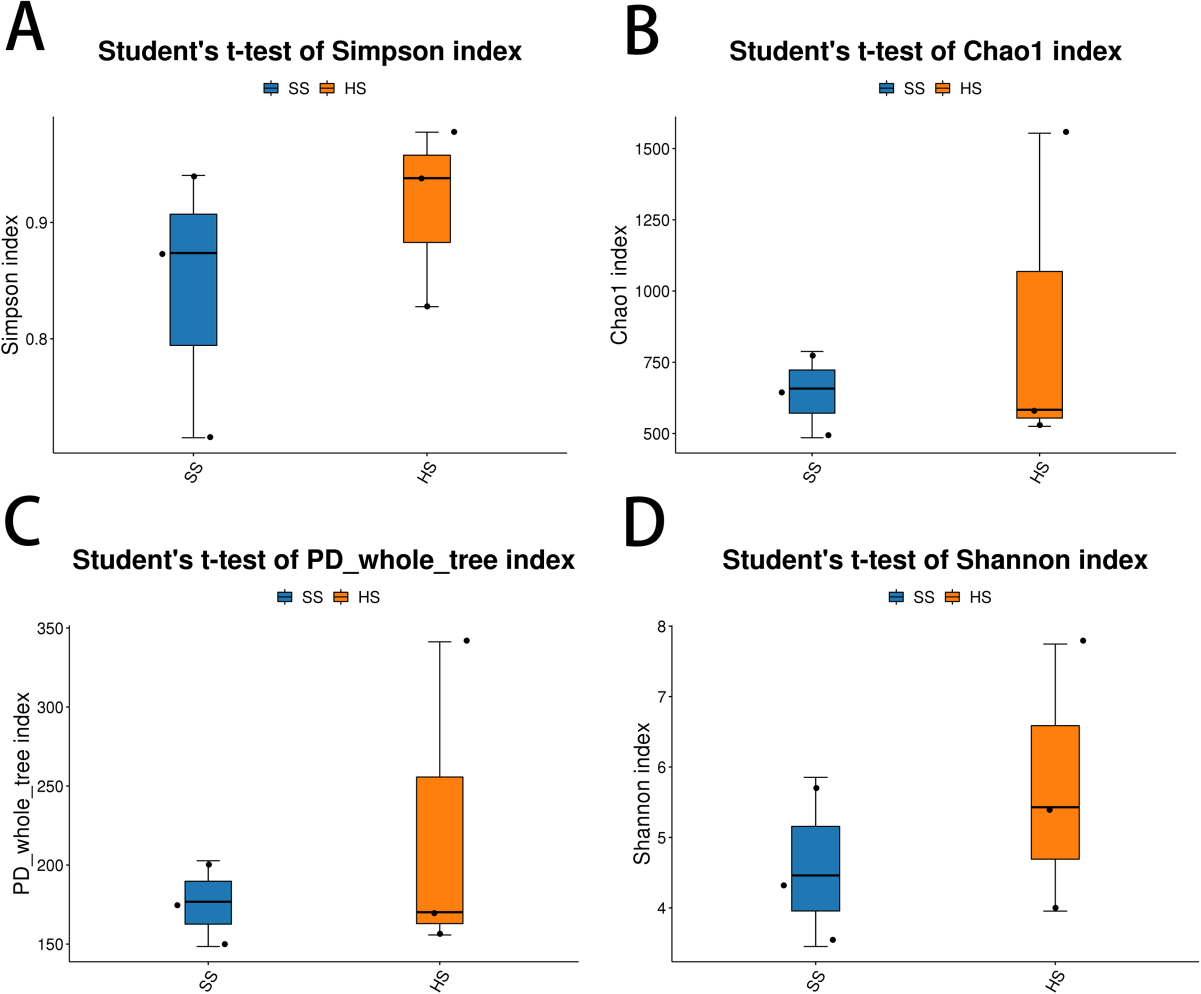
Alpha diversity indices of endophytic fungal communities in healthy (HS) and diseased (SS) *M. savatieri.* **(A)** Simpson index; **(B)** Chao1 index; **(C)** PD whole tree index; **(D)** Shannon index. Values are presented as mean ± SD (n = 3 biological replicates per group).*P < 0.05.

The Simpson index, which measures species evenness and dominance, was significantly higher in the HS group than in the SS group (**Figure 1A**), indicating a more evenly distributed endophytic fungal community in healthy plants. In contrast, the lower Simpson index in the SS group suggests that root rot infection may have led to the dominance of certain fungal taxa, reducing community evenness.

The Chao1 index, an estimator of species richness, also showed a marked decrease in the SS group compared to the HS group (**Figure 1B**). This result implies that healthy plants harbored a greater number of fungal species, while species richness was significantly reduced in diseased plants.

Phylogenetic diversity, assessed using the PD whole tree index, was substantially higher in the HS group (**Figure 1C**). This suggests that the endophytic fungal communities in healthy plants were not only more species-rich but also phylogenetically more diverse, potentially due to the loss of specific fungal lineages in response to disease.

Finally, the Shannon index, which integrates both species richness and evenness, was significantly elevated in the HS group (**Figure 1D**), further confirming that root rot disease severely compromised the overall diversity of endophytic fungal communities in *M. savatieri*.

Taken together, these results demonstrate that root rot infection leads to a significant reduction in the richness, evenness, and phylogenetic diversity of endophytic fungi associated with *M. savatieri*.

#### 3.1.3 Fungal community structure analysis

**Figure 2:**
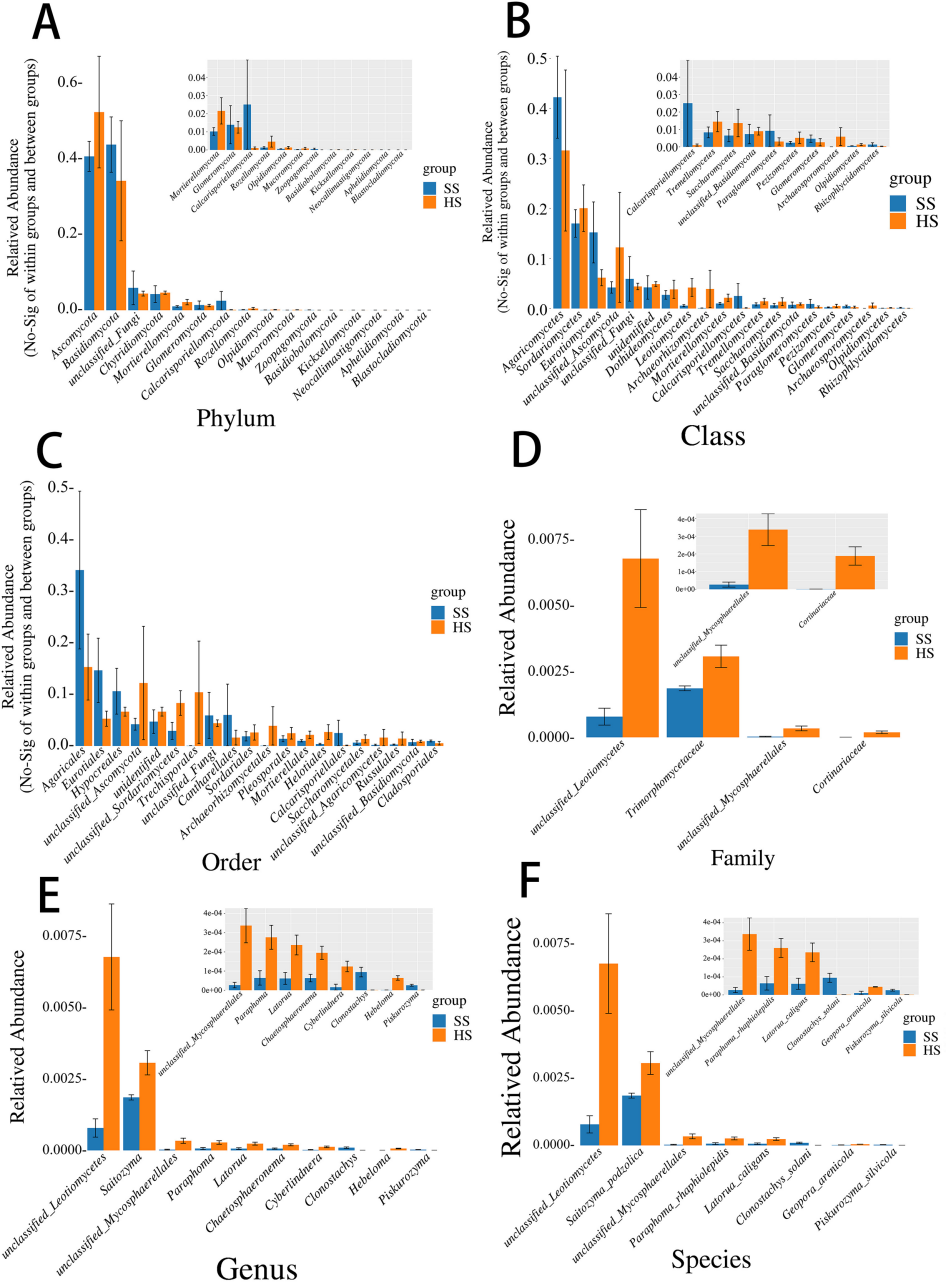
Bar chart of intergroup variance analysis at different taxonomic levels. **(A)** Phylum-level distribution of fungal communities; **(B)** Class-level distribution of fungal communities. **(C)** Order-level distribution of fungal communities; **(D)** Family-level distribution of fungal communities; **(E)** Genus-level distribution of fungal communities; **(F)** Species-level distribution of fungal communities. Data represent mean relative abundance ± SD (n = 3 biological replicates).

To assess the impact of root rot disease on the endophytic fungal communities of *M. savatieri*, rank-sum tests (ANOVA) were performed to compare community composition at multiple taxonomic levels: phylum, class, order, family, genus, and species (**Figure 2**). Structural differences between the healthy group (HS) and the diseased group (SS) were examined accordingly.

At the phylum level, Ascomycota and Basidiomycota were the dominant endophytic fungal groups in both HS and SS samples, with no statistically significant differences between the two groups.

At the class level, Agaricomycetes showed a higher relative abundance in the SS group, while Sordariomycetes were more prevalent in the HS group; however, these differences were not statistically significant. Other low-abundance fungal classes did not exhibit obvious group-specific patterns.

At the order level, Agaricales were more abundant in the SS group, while Eurotiales were relatively enriched in the HS group, suggesting that root rot may influence the distribution of specific fungal orders.

At the family level, unclassified_Lecanicillium showed a significantly higher relative abundance in the SS group, suggesting a potential association with disease occurrence. Trimorphomycetaceae also exhibited increased abundance in the SS group, indicating a possible ecological advantage under diseased conditions.

At the genus level, Saitozyma was significantly more abundant in the SS group compared to HS, and Paraphoma also showed higher relative abundance in SS samples, indicating that these genera may possess enhanced ecological adaptability in root rot-affected environments.

At the species level, both unclassified_Lecanicillium and Saitozyma podzolica were significantly more abundant in the SS group, implying a potential role in the pathogenesis or progression of root rot. Collectively, these findings demonstrate that root rot disease significantly alters the composition of endophytic fungal communities in *M. savatieri*, particularly at the family, genus, and species levels. Certain taxa, such as Saitozyma and Paraphoma, were markedly enriched in diseased plants and may be involved in the onset or development of root rot.

### 3.2 Comparative analysis of metabolites between healthy and root rot-infected *M. savatieri* plants

#### 3.2.1 Metabolomic analysis of healthy and diseased *M. savatieri* samples based on PCA and PLS-DA

**Figure 3:**
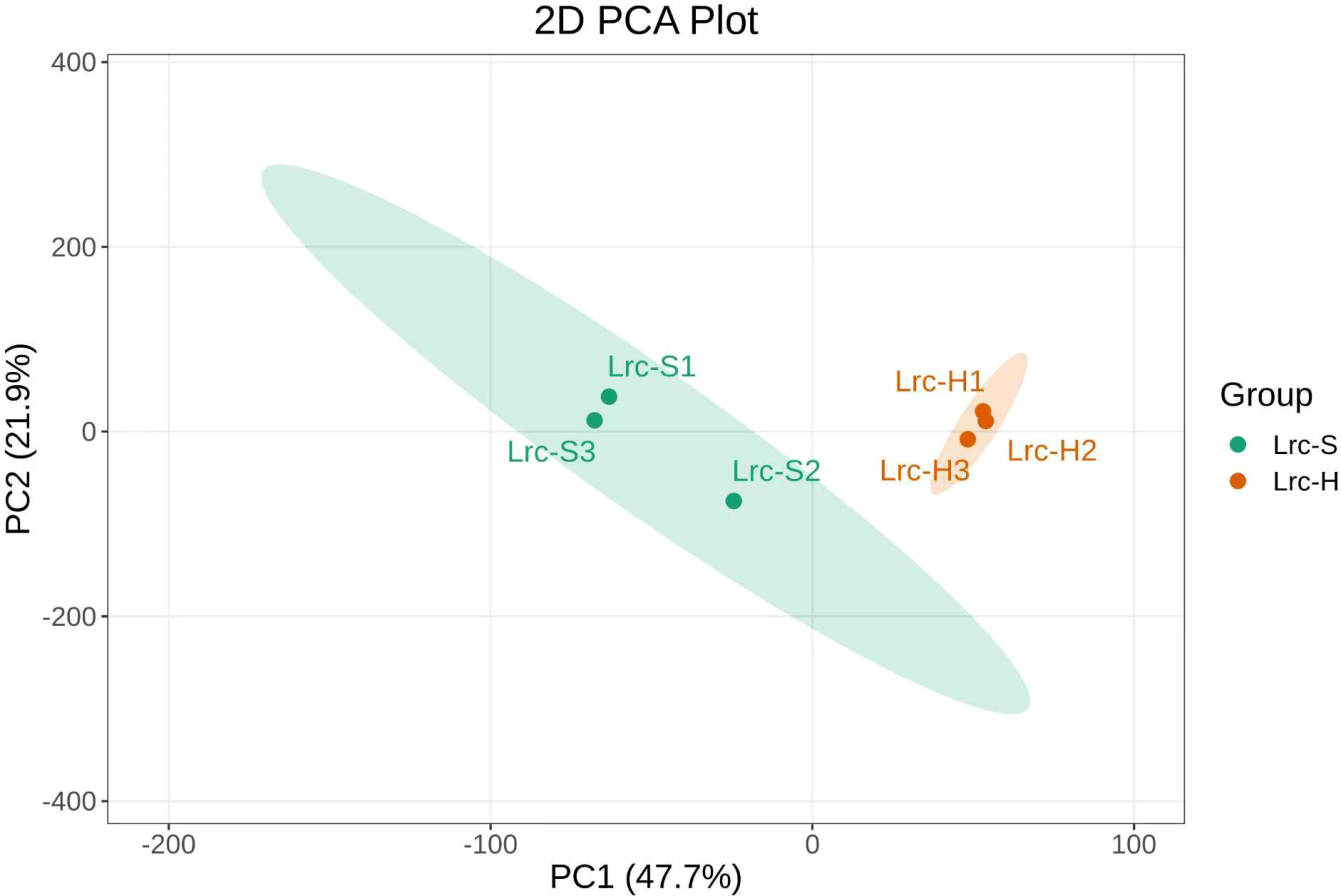
PCA plot of differential metabolites in water extracts between diseased and healthy *M. savatieri* plants. Each point represents one biological replicate (n = 3).

Principal Component Analysis (PCA) was employed to provide an overview of the metabolic differences between diseased and healthy *M. savatieri* samples. As shown in **Figure 3**, a clear separation trend was observed between the two groups based on their aqueous extract metabolite profiles. The first principal component (PC1) accounted for 47.7% of the total variance, indicating substantial differences in metabolite composition between healthy and diseased groups. The second principal component (PC2) explained 21.9% of the variance, suggesting relatively low variation among biological replicates within each group and a high degree of sample consistency.

**Figure 4:**
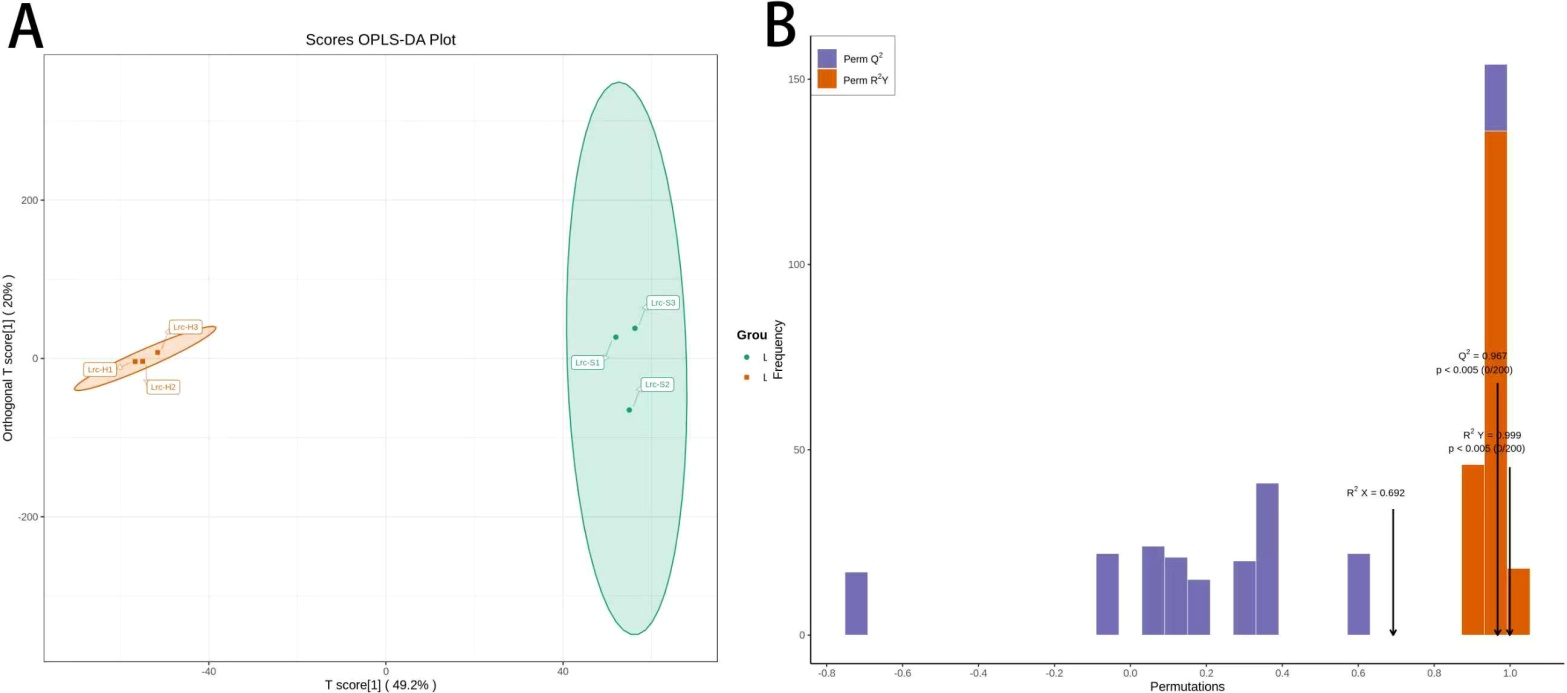
OPLS-DA analysis of diseased and healthy *M. savatieri*. **(A)**: OPLS-DA score plot; **(B)**: OPLS-DA validation Plot. Each point represents one biological replicate (n = 3).

To further enhance the separation between groups and minimize intra-group variation by eliminating noise and irrelevant variables, Partial Least Squares Discriminant Analysis (PLS-DA) was conducted. The score plot based on the PLS-DA model (**Figure 4A**) revealed a distinct separation between the healthy and diseased groups along the first predictive component (T score[1], 49.2%) and the first orthogonal component (Orthogonal T score[1], 20%). The tight clustering of samples within each group and clear inter-group discrimination demonstrate that the metabolomic profiles differ significantly between healthy and diseased *M. savatieri*, reflecting biologically meaningful phenotypic differences.

The PLS-DA model achieved a Q² value of 0.976, substantially exceeding the commonly accepted threshold of 0.9, thereby indicating excellent predictive performance and strong explanatory capacity. To evaluate the robustness and statistical validity of the model, a permutation test with 200 random permutations was conducted, and the results are presented in **Figure 4B**. The bar chart illustrates the distribution of R² (blue) and Q² (orange) values derived from the permuted models. Both the original R² and Q² values were significantly higher than those obtained from the permuted datasets, with p-values < 0.05, confirming the statistical significance of the model. Although the cross-validated Q² value (0.598) was lower than the model Q² (0.976), it still reflects acceptable predictive stability.

#### 3.2.3 Differential metabolite analysis

**Figure 5:**
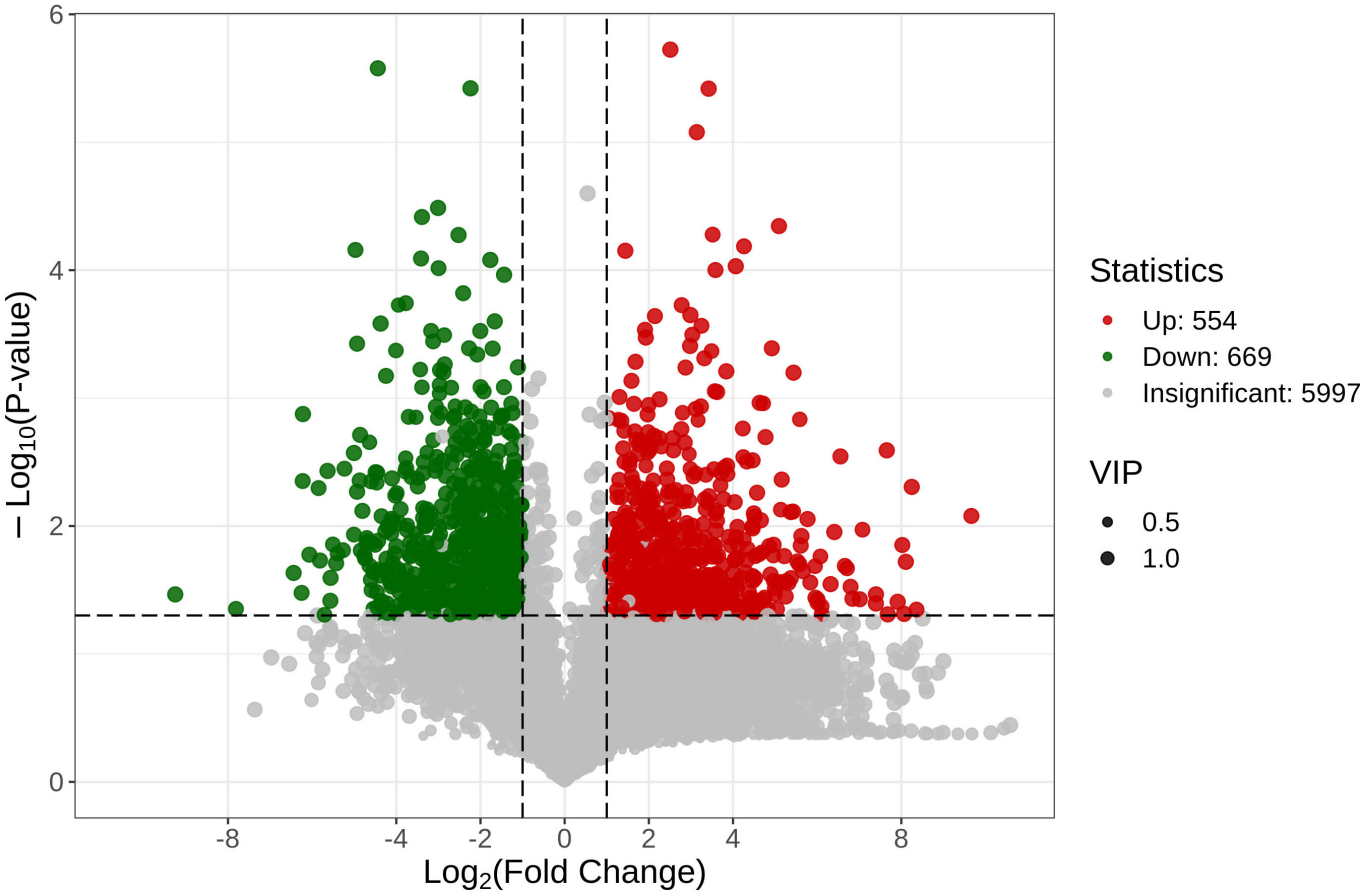
Volcano plots of differential metabolites between groups under different screening conditions.

Volcano plot analysis was performed to assess the differences in metabolite abundance between the diseased (Lrc-S) and healthy (Lrc-H) groups. The x-axis represents the log_2_(Fold Change) of metabolite abundance, while the y-axis indicates the -log_10_(p-value), reflecting the statistical significance. As shown in **Figure 5**, a total of 554 metabolites were significantly upregulated (marked in red), and 669 were significantly downregulated (marked in green), whereas 5,997 metabolites (gray) showed no significant change.

**Figure 6:**
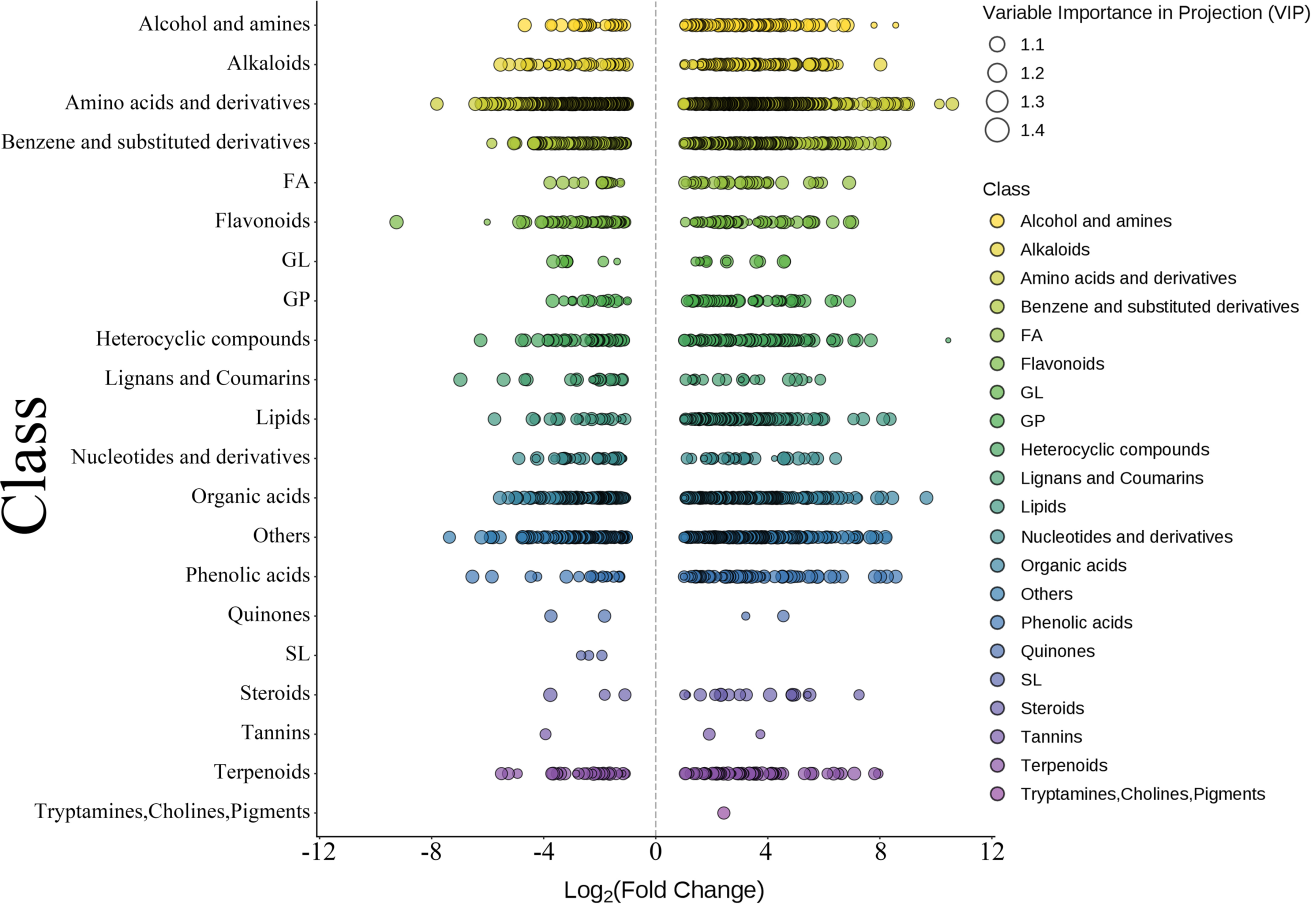
Scatter plot of differential metabolites. Data derived from n = 3 biological replicates per group.

The scatter plot of differential metabolites (**Figure 6**) further revealed that amino acids and their derivatives, flavonoids, organic acids, and alkaloids were significantly upregulated in the Lrc-H group, whereas sterols, terpenoids, and lipid-related metabolites were downregulated. The size of each bubble represents the Variable Importance in Projection (VIP) value, with larger bubbles indicating greater contributions to group discrimination.

These results suggest that enhanced amino acid metabolism may be associated with increased protein synthesis and energy metabolism. The enrichment of flavonoids and benzene-derived compounds could imply elevated antioxidant capacity. The accumulation of alkaloids and organic acids may reflect the activation of secondary metabolic pathways. In contrast, the reduction in sterol and terpenoid metabolites might indicate alterations in membrane composition or shifts in signaling molecule biosynthesis and regulation.

**Figure 7:**
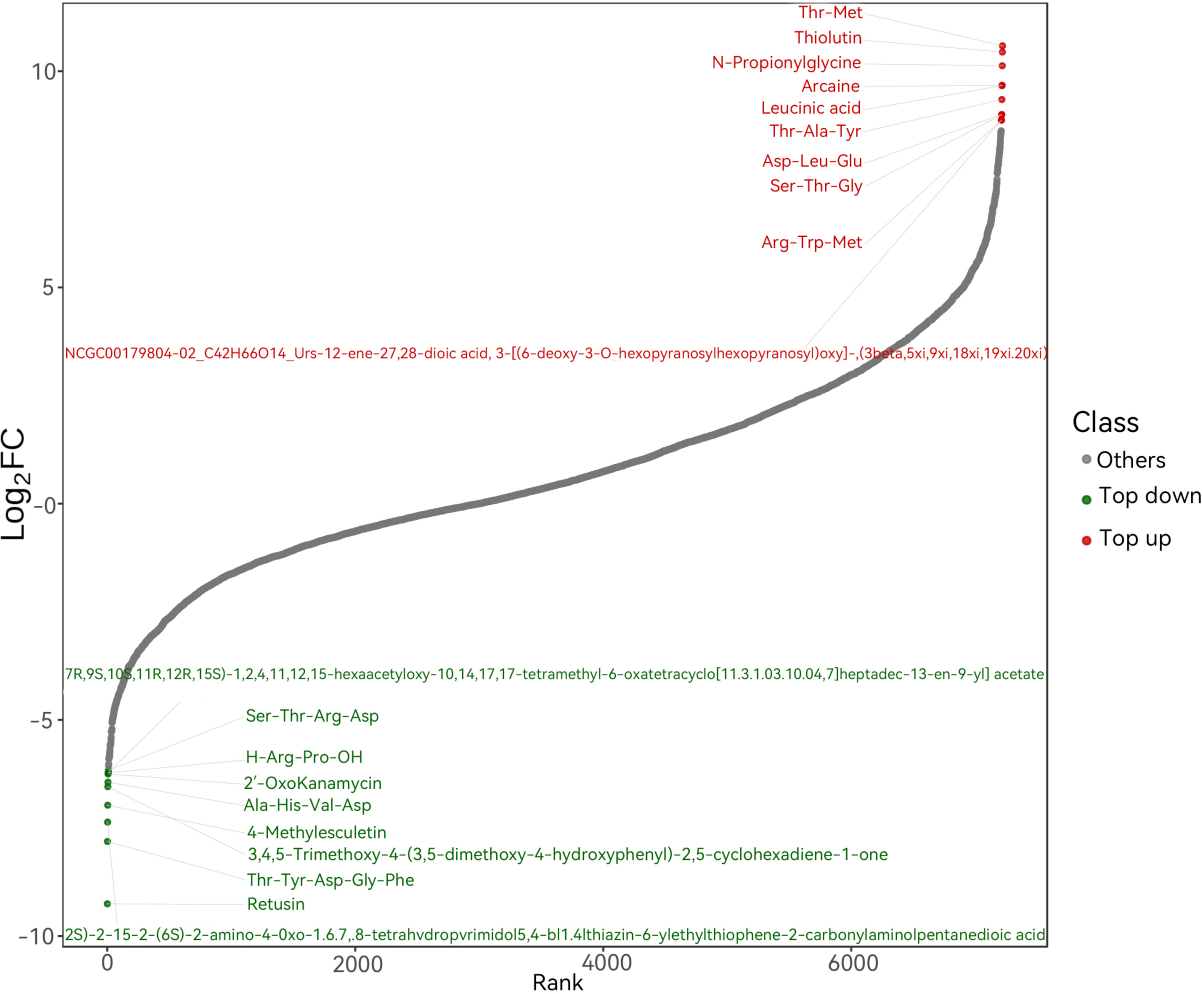
Dynamic plot of metabolite content differences. Data derived from n = 3 biological replicates.

A metabolite variation trend plot was generated based on Log_2_(Fold Change) analysis to visualize the abundance changes of metabolites between experimental groups (**Figure 7**). The x-axis represents the rank of metabolites, and the y-axis denotes the Log_2_(Fold Change) value. Upregulated metabolites are shown in red, downregulated metabolites in green, and non-significantly changed metabolites in gray.

Among the upregulated metabolites, threonine-methionine (Thr-Met), thiolutin, N-propionylglycine, arcainc (spermidine), and leucinic acid showed the most pronounced increases, indicating significant enrichment under the experimental conditions. These compounds are potentially involved in protein metabolism, antioxidant defense, and cellular signaling pathways.

In contrast, markedly downregulated metabolites included 7R,9S,10S,11R,12R,15S-1,2,4,11,12,15-hexaoxy-10,14,17,17-tetramethyl-6-oxatricyclo[11.3.1.0³,¹^0^.0^4^,^7^]heptadeca-13-en-9-yl acetate, H-Arg-Pro-OH (hydroxyarginine-proline dipeptide), 2’-oxoakamamycin, Ala-His-Val-Asp (tetrapeptide), and 4-methylsculetin (a coumarin derivative), suggesting a potential role in biosynthetic pathways, energy metabolism, and anti-inflammatory responses.

Overall, the significantly altered metabolites identified in this study are primarily involved in amino acid metabolism, nucleotide metabolism, lipid metabolism, and secondary metabolic pathways. These metabolic changes may contribute to the regulation of energy balance, redox homeostasis, and inflammatory processes under different experimental conditions. The observed shifts in key metabolites provide important insights into underlying metabolic regulatory mechanisms and offer a scientific basis for identifying potential biomarkers.

#### 3.2.4 Metabolic pathway analysis

**Figure 8:**
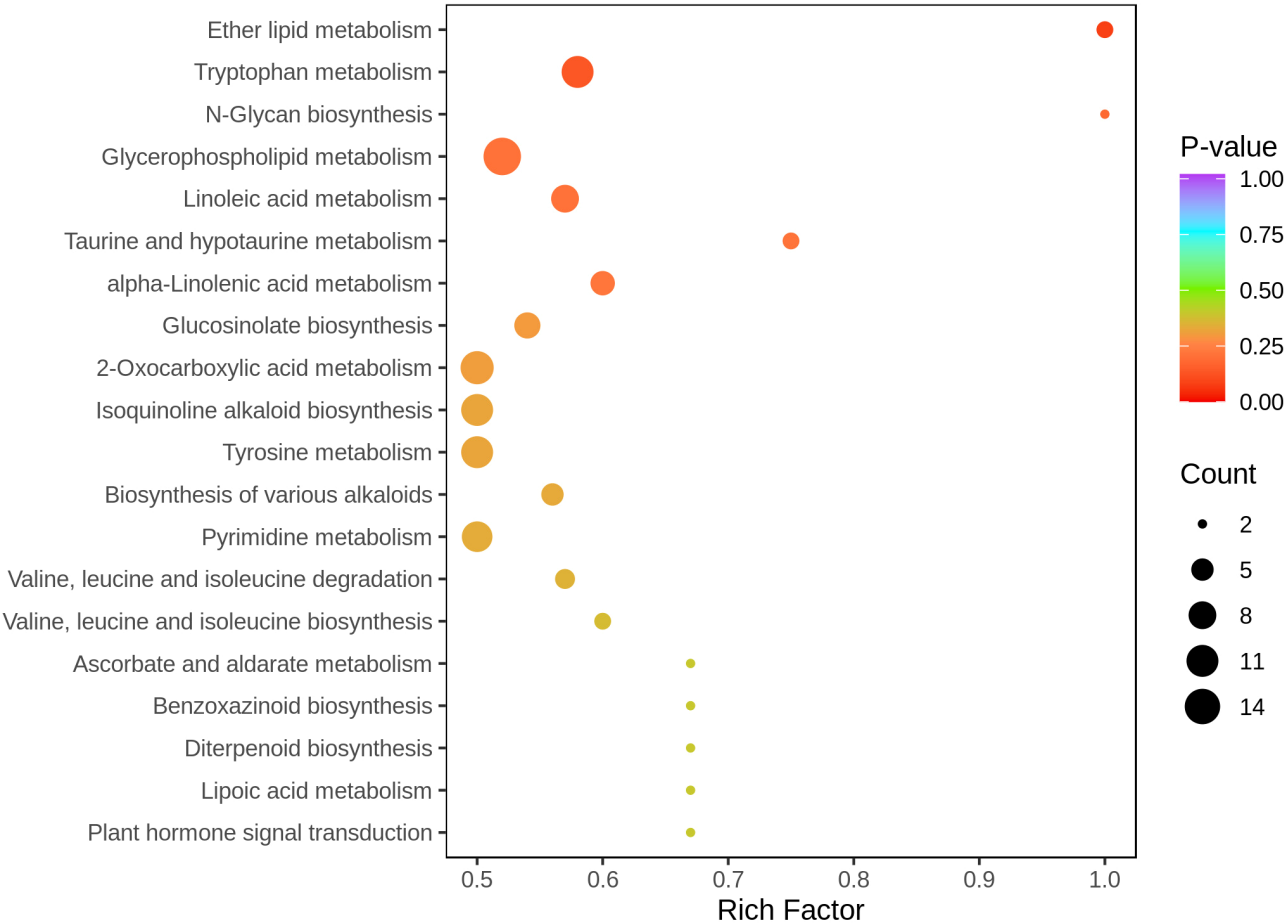
Pathway enrichment analysis of differential metabolites.

To elucidate the biological significance of the differential metabolites, pathway enrichment analysis was performed using the KEGG (Kyoto Encyclopedia of Genes and Genomes) metabolic pathway database. A KEGG pathway enrichment bubble plot was generated to visualize the results (**Figure 8**). In the plot, the rich factor represents the ratio of the number of differential metabolites to the total number of metabolites annotated in each pathway. The bubble size reflects the number of metabolites (Count), while the color gradient (from red to blue) indicates the statistical significance (P-value), with red denoting more significantly enriched pathways.

The analysis revealed that several pathways were significantly enriched, including ether lipid metabolism, tryptophan metabolism, N-glycan biosynthesis, glycerophospholipid metabolism, and linoleic acid metabolism. These pathways exhibited both high rich factors and low P-values, suggesting substantial alterations under different experimental conditions.

Specifically, ether lipid metabolism and glycerophospholipid metabolism, both essential components of membrane lipid dynamics, are implicated in maintaining cellular structural integrity, signal transduction, and inflammatory regulation. Tryptophan metabolism may be associated with neurotransmitter synthesis, immune modulation, and oxidative stress responses. Meanwhile, N-glycan biosynthesis is involved in protein glycosylation, potentially affecting cell-cell communication and immune responses.

Additionally, pathways such as taurine and hypoxanthine metabolism, alpha-linolenic acid metabolism, 2-oxocarboxylic acid metabolism, and isoquinoline alkaloid biosynthesis also showed varying degrees of enrichment. These are possibly involved in energy metabolism, redox balance, and the biosynthesis of plant-derived alkaloids, respectively.

In summary, the identified differential metabolites were mapped to multiple metabolic pathways related to lipid metabolism, amino acid metabolism, nucleotide metabolism, and secondary metabolism. These findings suggest that alterations in these metabolic processes may play crucial roles in physiological adaptations under different experimental conditions. Future studies combining metabolic flux analysis and molecular biology approaches are warranted to further explore the regulatory mechanisms of these pathways.

## 4 Discussion

In this study, we employed an integrated approach combining high-throughput sequencing and untargeted metabolomics to investigate the impact of root rot on *M. savatieri* from both microbial ecological and biochemical perspectives. Our results demonstrate that root rot infection induces profound disruptions at two interconnected levels: the endophytic fungal community and the plant’s metabolic network.

High-throughput sequencing revealed significant differences in the composition, abundance, and diversity of endophytic fungi between diseased (SS) and healthy (HS) groups, indicating that root rot systematically disturbs the plant’s microbial ecological balance. Specifically, the diseased group exhibited increased fungal abundance but reduced diversity, accompanied by an uneven species distribution. This pattern likely results from pathogenic fungal invasion or declining plant health, which allows certain fungal taxa to proliferate and disrupt the original microbial equilibrium. In contrast, healthy plants maintained higher alpha diversity and a more balanced community structure, reflecting a stable and coordinated endophytic fungal ecosystem. These findings are consistent with previous reports (Tan et al., 2017; Porras-Alfaro and Bayman, 2011; Trivedi et al., 2020), which have highlighted the critical role of endophytic fungal communities in plant health and disease resistance. When the microbial microecology is disrupted, the plant’s ability to resist environmental stressors may be weakened, thereby exacerbating disease incidence (Porras-Alfaro and Bayman, 2011). Therefore, maintaining or restoring the diversity and stability of root-associated and endophytic fungal communities may represent a promising approach for the biological control of plant diseases (Trivedi et al., 2020).

In this study, an untargeted metabolomic approach was employed to systematically characterize the chemical composition of *M. savatieri* and to explore metabolic alterations in response to root rot infection (Lrc-S vs. Lrc-H). The robustness of our analytical strategy was confirmed by multivariate statistical models; both Principal Component Analysis (PCA) and Partial Least Squares Discriminant Analysis (PLS-DA) revealed a distinct and reliable metabolic separation between healthy and diseased samples. The metabolic reprogramming associated with root rot was characterized by significant fluctuations in specific compound classes. Healthy *M. savatieri* plants exhibited a notable enrichment of amino acids and their derivatives, benzenoid compounds, and organic acids. These classes are widely recognized for their involvement in enhancing plant antioxidant capacity and regulating stress responses. For instance, the accumulation of amino acid derivatives is frequently linked to the activation of defense pathways under biotic stress. Concurrently, the enrichment of flavonoids—a subset of the identified benzenoid compounds—in healthy plants is particularly significant. Flavonoids are established natural antioxidants commonly associated with plant defense responses, potentially contributing to redox regulation and defense-related signaling during pathogen challenge (Anzano et al., 2022; Lu and Xia, 2025). This suggests that healthy plants maintain a heightened state of metabolic readiness, which may be crucial for effective pathogen resistance. Conversely, the onset of disease was marked by the downregulation of sterols, terpenoids, and other lipid-related compounds. Sterols and lipids are fundamental components of cell membranes, crucial for maintaining structural integrity and facilitating signal transduction pathways. The observed reduction in these metabolites in diseased samples indicates that root rot pathogenesis may be associated with the disruption of membrane homeostasis (Der et al., 2024). This alteration in membrane composition could impair the plant’s ability to perceive pathogen signals and mount an effective physiological defense, potentially accelerating disease progression (Seth et al., 2024). In conclusion, while our metabolomic profiling provides a highly sensitive and comprehensive view of the plant’s response to root rot, it is crucial to acknowledge its inherent limitations. The interpretations presented here are based on metabolite annotation and pathway enrichment, which, although consistent with reported defense-related functions, represent associative patterns rather than direct functional validation. The observed upregulation of compounds like flavonoids and amino acid derivatives should therefore be regarded as consistent with, but not definitive proof of, enhanced immunity.

Pathway enrichment analysis revealed significant perturbations in several metabolic pathways in response to root rot infection, including ether lipid metabolism, tryptophan metabolism, glycerophospholipid metabolism, and N-glycan biosynthesis. These findings provide valuable insights into the biochemical strategies employed by *M. savatieri* during pathogen stress. The enrichment of ether lipid and glycerophospholipid metabolism pathways is particularly noteworthy. As crucial components of cell membranes and precursors to plant hormones, these lipid classes play fundamental roles in energy storage and intracellular signaling (Kuźniak and Gajewska, 2024). The observed alterations suggest that membrane remodeling may be a central feature of the plant’s response to root rot. Given the importance of membrane integrity in pathogen recognition and defense signal propagation, these pathways warrant further investigation. Future studies should aim to elucidate how targeted regulation of lipid metabolism could enhance plant resistance to soil-borne pathogens such as those causing root rot. The significant enrichment of tryptophan metabolism aligns with its established role as a central hub for producing diverse secondary metabolites with defensive functions. Through enzymatic catalysis, tryptophan serves as a precursor for multiple bioactive compounds, including the plant hormone auxin, various pigments, and flavonols (Wang et al., 2025). Beyond its role as a metabolic precursor, tryptophan itself contributes to stress tolerance by modulating hormone biosynthesis, enhancing antioxidant enzyme activity, and regulating stress-responsive gene expression (Frerigmann et al., 2016). The enrichment of this pathway in our dataset suggests its potential involvement in coordinating defense responses against root rot. Future research should investigate the dynamic accumulation patterns of tryptophan-derived metabolites throughout disease progression, with particular attention to their roles in early pathogen recognition and the activation of downstream defense mechanisms.

The enrichment of the N-glycan biosynthesis pathway points to the potential involvement of protein glycosylation modifications in M. savatieri’s defense response. This process, which involves the transfer of oligosaccharide chains to specific amino acid residues of target proteins under the catalytic action of glycosyltransferases (Jiang et al., 2025), is increasingly recognized as a critical regulator of plant immunity. N-glycosylation plays a vital role in cell–cell communication and immune recognition during plant-pathogen interactions (Nagashima et al., 2018). The activation of this pathway in our study suggests that glycoprotein-mediated signaling may contribute to the plant’s ability to perceive and respond to pathogen invasion. Future investigations should focus on the functional characterization of specific N-glycans, particularly their potential applications in root rot management and their mechanistic roles in pathogen recognition and immune activation.

Emerging evidence indicates that root rot-induced alterations in the endophytic microbial community may compromise the disease resistance and ecological adaptability of *M. savatieri* (Tan et al., 2022). This observation highlights the importance of considering the plant holobiont—the plant host together with its associated microbiota—in disease studies. Future research strategies should include approaches aimed at enhancing rhizosphere microbial diversity as a means to promote plant health and resilience against pathogens. The current findings, derived from integrated differential metabolite analysis and metabolic pathway enrichment, align with this SoilTech perspective by providing a deeper understanding of the biochemical responses in M. savatieri under disease stress. These results not only reveal potential biomarkers of infection status but also suggest therapeutic targets for further pharmacological and agricultural interventions (Sun et al., 2025). As highlighted by Risoli et al. (2026), integrating such metabolite-level insights with soil microbiome management strategies could pave the way for more effective, system-oriented approaches to disease control in medicinal plant cultivation.

It should also be acknowledged that the present study focused exclusively on aerial tissues for both microbiome and metabolomic profiling. Although this approach aligns with the pharmacological significance of the aerial parts of M. savatieri, root rot is fundamentally a root-localized disease. Consequently, the absence of paired root and rhizosphere samples limits our ability to distinguish between localized infection processes and systemic plant responses. Future investigations should therefore integrate paired analyses of root, rhizosphere soil, and aerial tissues to elucidate the spatial dynamics of disease response and to clarify how belowground microbial disruptions translate into aboveground metabolic and community-level shifts.

Furthermore, high-throughput sequencing data offer compelling evidence that root rot disrupts the diversity and uniformity of endophytic fungal communities, ultimately impacting plant health. To build upon these observations, future research should integrate metabolic flux analysis with molecular biology techniques to explore the precise biological functions of the aforementioned metabolic pathways. Such integrated approaches will provide a theoretical foundation for developing novel plant disease control strategies, potentially targeting both plant metabolism and the beneficial modulation of associated microbial communities.

## Data Availability

The original contributions presented in the study are publicly available. This data can be found here: NCBI, PRJNA1462741.
