# Correction: Integrated analysis of endophytic fungal communities and metabolites reveals root rot-induced disruptions in *Monochasma savatieri*

**DOI:** 10.3389/fpls.2026.1888132

**Published:** 2026-06-10

**Authors:** Yuyin Zhang, Hua Dou, Chenlu Fan, Xuyu Chen, Jianhe Wei

**Affiliations:** 1 Hainan Provincial Key Laboratory of Resources Conservation and Development of Southern Medicine, Hainan Branch of Institute of Medicinal Plant Development, Chinese Academy of Medicinal Sciences & Peking Union Medical College, Haikou, Hainan, China; 2 Key Laboratory of Bioactive Substances and Resources Utilization of Chinese Herbal Medicine, Ministry of Education & National Engineering Laboratory for Breeding of Endangered Medicinal Materials, Institute of Medicinal Plant Development, Chinese Academy of Medical Sciences & Peking Union Medical College, Beijing, China

**Keywords:** endophytic fungi, *M. savatieri*, metabolomics, plant disease, root rot

The funders “National Key R&D Program of China”, “2022YFC3501504”, “the CAMS Innovation Fund for Medical Sciences (CIFMS)”, “2021-I2M-1-032”, “the National Administration of Traditional Chinese Medicine”, “GZY-KJS-2023-009”, and “the Talents Development Bureau of the CPC Hainan Provincial Committee”, “HNYT20240003” were erroneously omitted from the **Funding** statement.

The original version of this article has been updated.

